# Intracardiac Origin of Heart Rate Variability, Pacemaker Funny Current and their Possible Association with Critical Illness

**DOI:** 10.2174/157340313805076359

**Published:** 2013-02

**Authors:** Vasilios E Papaioannou, Arie O Verkerk, Ahmed S Amin, Jaques MT de Bakker

**Affiliations:** 1 Democritus University of Thrace, Alexandroupolis University Hospital, Intensive Care Unit, Greece; 2 Department of Anatomy, Embryology and Physiology; 3 Department of Clinical and Experimental Cardiology, University of Amsterdam, Heart Failure Research Center, Academic Medical Center, The Netherlands

**Keywords:** Endotoxin, funny current, heart rate, heart rate variability, ivabradine, sepsis, sinoatrial node.

## Abstract

Heart rate variability (HRV) is an indirect estimator of autonomic modulation of heart rate and is considered a risk marker in critical illness, particularly in heart failure and severe sepsis. A reduced HRV has been found in critically ill patients and has been associated with neuro-autonomic uncoupling or decreased baroreflex sensitivity. However, results from human and animal experimental studies indicate that intracardiac mechanisms might also be responsible for interbeat fluctuations. These studies have demonstrated that different membrane channel proteins and especially the so-called ‘funny’ current (I_f_), an hyperpolarization-activated, inward current that drives diastolic depolarization resulting in spontaneous activity in cardiac pacemaker cells, are altered during critical illness. Furthermore, membrane channels kinetics seem to have significant impact upon HRV, whose early decrease might reflect a cellular metabolic stress. In this review article we present research findings regarding intracardiac origin of HRV, at the cellular level and in both isolated sinoatrial node and whole *ex vivo* heart preparations. In addition, we will review results from various experimental studies that support the interrelation between I_f_ and HRV during endotoxemia. We suggest that reduced HRV during sepsis could also be associated with altered pacemaker cell membrane properties, due to ionic current remodeling.

## INTRODUCTION

Healthy state exhibits some degree of stochastic variability in physiologic variables, such as heart rate (i.e. heart rate variability-HRV). This variability is a measure of complexity that accompanies healthy systems and has been suggested as responsible, for their greater adaptability and functionality related to pathologic systems [1]. Loss of such variability means loss of complexity that accompanies cardiovascular disease and critical illness [2,3], while it is associated with increased mortality rate after acute myocardial infarction [4], sepsis and multiple organ dysfunction syndrome (MODS) [3,5]. 

Studying physiological signals of critically ill patients, such as heart rate, can easily identify ‘hidden’ information concerning inherent dynamics and overall variability within time series [2]. Recognition that physiologic time series contain hidden information related to an extraordinary complexity that characterizes physiologic systems defies traditional mechanistic approaches based on conventional biostatistical methodologies and has fueled growing interest in applying techniques from statistical physics for the study of living organisms [1,2]. This effort has boosted research on heart rate and blood pressure dynamics through standardization of different signal processing techniques, frequency and duration of measurements and signal quality assessment, and has stimulated the development of more accurate diagnosticand prognostic indices in cardiovascular diseases [6]. Furthermore, a number of international databases of heart rate signals have been developed with free access from different investigators, such as the Web Site Physionet (www.physionet.org) [6]. In conclusion, the combination of structural indices such as the left ventricular ejection fraction (LVEF) with autonomic function indices derived from heart rate variability analysis has been recently proposed as the state-of-the-art method for risk assessment among patients with acute myocardial infarction or severe congestive heart failure [7].

In this review article we will describe the basic methods for assessment of HRV, discuss potential mechanisms responsible for its generation at the intracardiac level and summarize the most recent and significant studies, from both basic and clinical research, which investigate the potential effects of cardiovascular diseases and sepsis upon HRV alterations. Finally, we will present research data concerning the association between critical illness, HRV and the ‘funny’ current (I_f_) that is responsible, among others, for the generation of action potentials (APs) by pacemaker cells in the sinoatrial node (SAN).

## MEASUREMENT OF HRV

Heart rate variability describes variations in both instantaneous heart rate and RR intervals. HRV is considered an indirect measure of autonomic regulation of cardiac activity, and can reflect the coupling between the autonomic nervous system (ANS) and the sinoatrial node [3]. In 1996, the Task Force of the European Society of Cardiology and the Northern American Society of Pacing and Electrophysiology published guidelines regarding standardization of nomenclature, specification of methods of measurement, definition of physiological and pathophysiological correlates, description of clinical applications and identification of different areas for future research [8]. 

The RR variations may be evaluated by two methods derived from: 1) time domain, and 2) frequency domain. 

### Methods of Measurement

#### Time Domain Methods

Time domain methods determine heart rate or RR intervals in continuous electrocardiographic (ECG) recordings. Each QRS complex is detected and the normal-to-normal intervals (all intervals between adjacent QRS complexes) are calculated. Other time domain variables include the mean normal-to-normal interval, the mean heart rate or the difference between the longest and the shortest interval as well. There are also more complex statistical methods being used, particularly from heart rate signals being recorded for more than 24 hours. The simplest from these metrics is the standard deviation of the normal-to-normal intervals (SDNN), which is the square root of the variance. However, it should be emphasized that the SDNN variable becomes less accurate by shorter the monitoring periods since it depends on the duration of RR interval series and its duration must be standardized (e.g. 5 min or 24 hours) [8]. The most commonly used time domain methods are the square root of the mean squared differences of successive intervals (RMSSD), the number of interval differences of successive intervals greater than 50 ms (NN50) and the proportion derived from dividing NN50 by the total NN intervals (pNN50) [8].

#### Frequency Domain Methods 

Akselrod *et al.* [9] introduced in 1981 power spectrum analysis of heart rate fluctuations in order to quantify beat-to-beat cardiovascular control. Power spectrum density (PSD) analysis provides the basic information of how power (variance, msec^2^/Hz) distributes as a function of frequency. Spectral analysis of heart rate signals provides their power spectrum density and displays in a plot the relative contribution (amplitude) of each frequency, after application of a Fast Fourier transformation (FFT) to the raw signal. This plot includes at least three frequency peaks. Fast frequency periodicities (high frequency, HF), in the range 0.15-0.4 Hz, are largely due to the influence of the respiratory phase on vagal tone. Low-frequency periodicities (LF), in the range of 0.04-0.15 Hz, are produced by baroreflex feedback loops, affected by both sympathetic and parasympathetic modulation of the heart. Very low frequency periodicities (VLF), i.e. less than 0.04 Hz, have been variously ascribed to modulation by chemoreception, thermoregulation and the influence of vasomotor activity, which is related, between others, to the renin-angiotensin-aldosterone system (RAS) [8-10]. The area under the power spectral curve in a particular frequency band (power) is considered to be a measure of heart rate variability at that frequency. The ratio LF/HF reflects sympathovagal balance whereas normalized units (nu) of both LF and HF (LF/total power and HF/total power, respectively) indicate heart rate variability in specific bands irrespectively of total variability of the whole signal [8]. In a double logarithmic plot of power versus frequency, their relation follows a straight line with a slope defined as β. This relation is known as the power law, whereas in normal subjects, β slope or exponent is close to -1 [8,11]. 

### Extracardiac Origin of HRV

The LF component of HRV is probably the most contentious aspect with respect to cardiovascular variability. There are two opposing theories in the literature proposing different potential origins: 1) the central oscillator theory, and 2) the baroreflex feedback loop theory [12,13]. According to the first theory, it is believed that LF oscillations reflect sympathetic tone and are generated by the brain stem circuits. In cats, Montano* et al.* [12] analyzed the discharges of single sympathetic neurons located in the rostral ventrolateral medulla (RVLM) and caudal ventrolateral medulla (CVLM). They observed activity at 0.12 Hz, which was positively correlated with heart rate and blood pressure variability. As the above oscillations remained after sino-aortic and vagal resection, it was assumed that the central nervous system is able to generate such oscillations. 

The second, more accepted theory is baroreflex feedback loop model [13], where a change in blood pressure is sensed by arterial baroreceptors, resulting in heart rate adjustment through the central nervous system and via both the fast vagal action and the slower sympathetic action. At the same time, baroreceptors induce a slow sympathetic withdrawal from the vessels. The delay in the sympathetic branch of the baroreflex in turn determines a new oscillation, which is sensed by the baroreflex and induces a new oscillation in heart rate. It has been also proposed that the LF oscillation arises from the interaction of slow sympathetic and fast vagal responses, where baroreflex buffering of the slow respiratory induced blood pressure oscillations results in resonant low frequency oscillations, due to the delay in the slow conducting sympathetic loop of the baroreflex [14].

#### Power Law and HRV

**Fig. (1):**
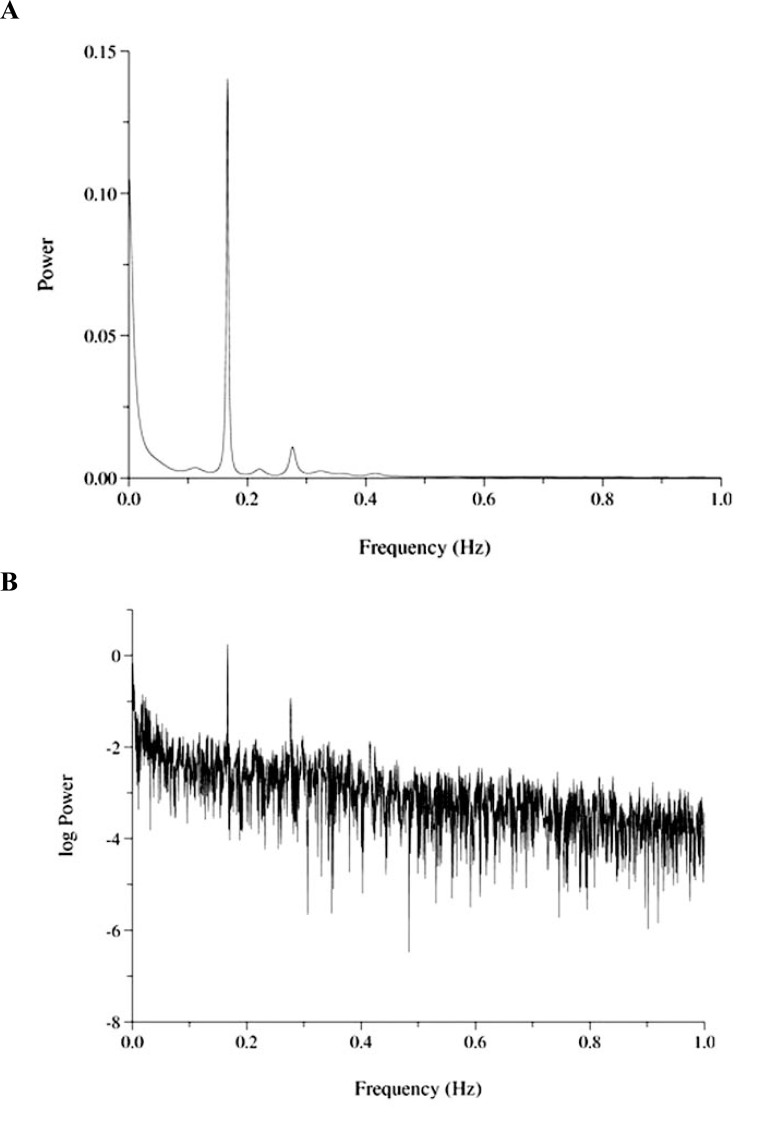
Fast Fourier transformation (FFT) of a heart rate time series **(A)** and its power law distribution **(B)**. The spectrum of the heart rate time series displays three peaks mainly at the lower frequencies (**A**) whereas the log transformation of the power of the signal gives rise to a more or less linear relation (**B**). If we apply log transformation of the frequency as well (not shown here), we will have a more smooth linear relation with a slope β. Data have been downloaded from the free-download website physionet (www.physionet.org).

In terms of power spectrum density, the major component of HRV occurs at frequencies below 0.04 Hz, where its power spectrum exhibits a power law behavior. Fluctuations of a variable can be characterized by its probability density distribution. A way of estimating its characteristics is the construction of a histogram after normalization, so that the area under it will be equal to one. Often, this distribution N(x) of a variable x follows the power law form: N(x) = k*x^-d ^meaning that the relative frequency of a value x is proportional to x raised to the power of –d, whereas a constant multiplicative factor k (usually different from unity) must be incorporated in the equation. If we plot the logarithms of this relationship we have a linear equation: log (N) = log (k)-d*log(x), whereas d is the negative slope of a straight line fit to N. This slope is the β slope or exponent. (Fig. **1A** & **B**) [15,16].

Power law distribution behaves differently than Gaussian distributions. In power law distributions, tails are very long (long-tail distribution), representing the relative frequency of occurrence of large events. This means that the probability of large or rare events is much higher compared with a Gaussian. Power laws describe dynamics that have a similar pattern of change at different scales and they are called ‘scale invariant’. On the contrary, Gaussians are characterized by typical values, such as those corresponding to their peaks [16]. Moreover, the power law describes a time series whose pattern of variation is statistically similar regardless of its size. Magnifying or shrinking the scale of the signal reveals the same relationship, a property that has been called ‘self-similarity’ and is a fundamental characteristic of fractals [16,17]. These structures appear similar at different scales of magnification, meaning that in cases of different time series (e.g., heart rate) this property of self-similarity indicates the presence of long-term correlations across multiple temporal scales. Origin of power law behavior in healthy state is still not known, whereas decrease or more negative values of β slope have been described in different situations, such as myocardial infarction [11] and severe sepsis [5]. 

### Intracardiac Origin of HRV

Except for extracardiac mechanisms, recent evidence from *in vitro* and *ex vivo *experimental studies suggests that HRV might also depend on factors intrinsic to cardiac tissue, whereas fluctuations in spontaneously beating rate of single cardiac cells , here defined as beating rate variability (BRV), seem to follow a power law behavior [18-21]. Moreover, different clinical studies in heart transplant recipients have found evidence for heart rate fluctuations originating from the heart itself [22,23]. Bernardi *et al.* [22] studied intrinsic mechanism regulating HRV in both transplanted and intact heart during exercise. They found that that at peak exercise a non-autonomic mechanism, probably intrinsic to the heart muscle, may determine heart rate fluctuations in synchrony with ventilation, in transplanted as well as in intact hearts. Hrushesky and colleagues [23] quantified respiratory sinus arrhythmia (RSA) and found that individuals with a transplanted heart had resting RSA values similar to healthy subjects. 

#### Cardiac Pacemaking Mechanism

**Fig. (2):**
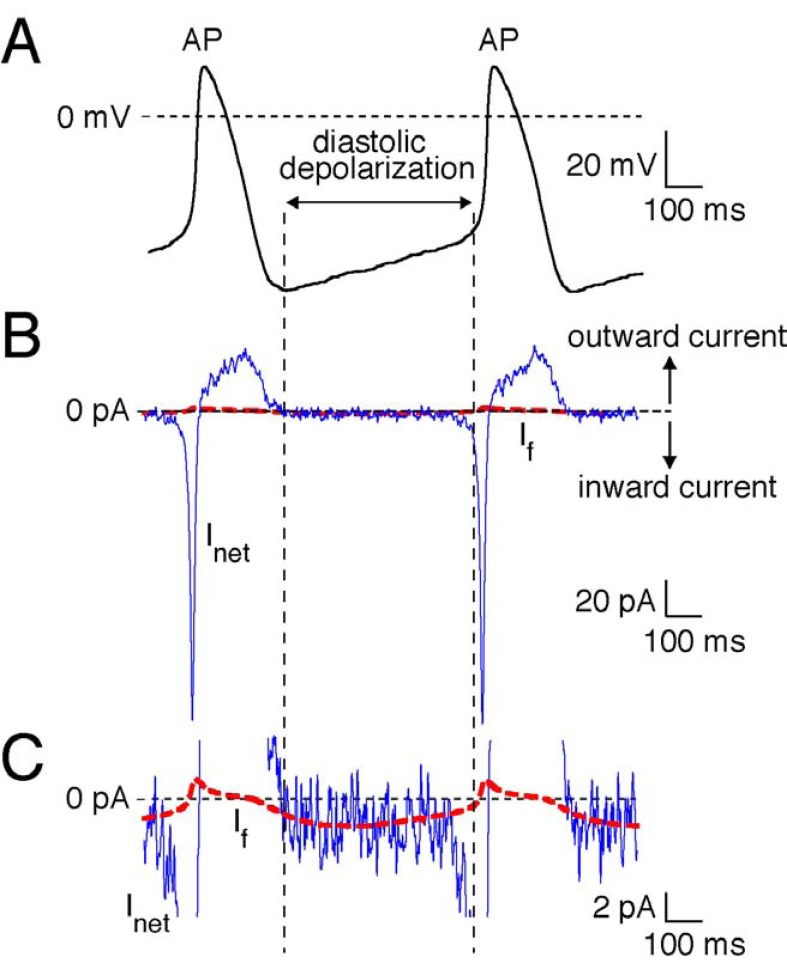
Experimentally recorded action potential (AP) of a single human SAN pacemaker cells. **A**. For cell isolation and recording procedure, see Ref 25. **B**. Associated net membrane current (I_net_, blue line) calculated from I_net_=- C_m_×dV_m_/dt, where C_m_ and V_m_ denote membrane capacitance and membrane potential, respectively. Note the small inward current underlying the slow diastolic depolarization. **C**. Computed I_f_ (solid red line). The time course of I_f_ was reconstructed using the V_m_ values of the recorded action potentials and a first-order Hodgkin–Huxley type kinetic scheme based on voltage clamp data from human SAN pacemaker cells. For details, see Ref 24.

**Fig. (3):**
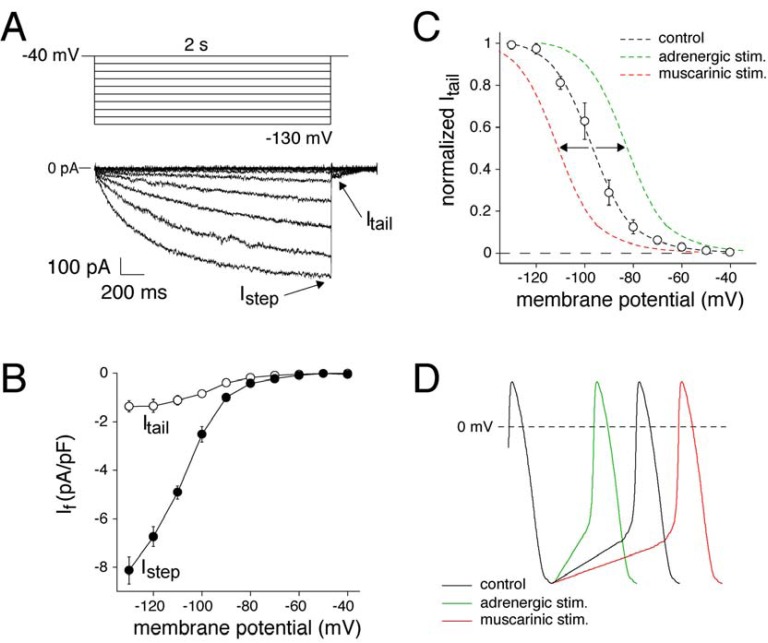
Time and voltage dependence of the hyperpolarization-activated ‘funny’ current (I_f_) in a single human SAN pacemaker cell. **A**. Voltage clamp protocol (top) and associated I_f_ current traces (bottom). Arrows indicate I_f_ step and tail current (I_step_ and I_tail_, respectively). I_f_ current traces were obtained by digital subtraction of current traces recorded in the absence and presence of 2 mmol/L external Cs^+^. For details, see Ref 25. **B**. Average current-voltage relationship of I_step_ and I_tail_, respectively, of three isolated human SAN pacemaker cells. **C**. Steady-state activation curves of If. Black dashed line is the Boltzmann fit to the experimental data of three human SAN cells; Green dashed line is a schematic representation of adrenergic effects on the steady-state activation curve; Red dashed line is a schematic representation of muscarinic effects on the steady-state activation curve. **D**. Schematic representation of adrenergic and muscarinic effects on the SAN AP.

Cardiac pacemaking is a basic physiological function required to match cardiac performance to metabolic demand. In the mammalian heart, it is accomplished by pacemaker cells in the sinoatrial node region, which is located in the right atrium. The SAN consists of specialized cells that exhibit spontaneous electrical activity, i.e., generating of recurring action potentials due to slow diastolic depolarization (Fig. **2A**). Diastolic depolarization is due to a small net inward current across the cell membrane ((Fig. **2B**), blue solid line), which is the result of the “membrane clock”, a complex interaction of multiple time- and voltage-dependent inwardly and outwardly directed ion currents, including the ‘funny’ current, I_f_ ((Fig. **2C**), red dashed line) [24, 25]. I_f_ is called ‘funny’ current because it is activated upon hyperpolarization and is carried by Na^+^ and K^+^ transport. (Fig. **3A**) shows a typical example recorded in a SAN cell isolated from a human heart. Typically, I_f_ becomes larger and activated more rapidly at increasingly negative potentials. (Fig. **3B**) shows the average I_step_, i.e., the current measured at the end of the 2 sec hyperpolarizing voltage clamp step, and I_tail_ , i.e. the currents measured by stepping back to the holding potential of –40 mV, of 3 human SAN cells. Voltage-dependence of I_f_ activation is analyzed by plotting normalized I_tail_ amplitude against the preceding voltage steps, and is shown in (Fig. **3C**). The average half-maximal activation voltage (V_1/2_) and slope factor of the Boltzmann fit to the data (black dashed line) was -96.9±2.7 and -8.8±0.5 mV, respectively. Molecular characterization has demonstrated that I_f_ is encoded by a family of 4 genes, termed *H*yperpolarization-activated *C*yclic *N*ucleotide (HCN) genes. I_f_ observed in human SAN is likely carried by HCN4 channel proteins because they are the dominant expressed isoforms in human SAN [24-26]. I_f_ is an important target of heart rate regulation from the autonomic nervous system [24]. In particular, β-adrenergic stimulation through its activation of adenylyl cyclase elevates cAMP, which then binds to the cytoplasmatic tails of I_f _channel and shifts the voltage-dependency of activation to more positive potentials. (Fig. **3C**) shows a schematic drawing of such an effect (green dashed line). As a consequence, there is an acceleration of spontaneous electrical activity through an increase of the rate of diastolic depolarization as schematically drawn in (Fig. **3D**), green solid line. On the contrary, acetylcholine via muscarinic receptors inhibits adenylyl cyclase and shifts the I_f_ voltage-dependency of activation to more negative potentials ((Fig. **3C**), red dashed line), thereby decreasing the diastolic depolarization rate ((Fig. **3D**), red solid line) [24-26]. 

In the last decade, spontaneous Ca^2+^ releases from the sarcoplasmic reticulum (SR) have been recognized as an additional mechanism for SAN cell pacemaker activity: the ‘‘Ca^2+^ clock’’ [27]. Lakatta and colleagues [27] showed that spontaneous local subsarcolemmal Ca^2+ ^released from the sarcoplasmatic recticulum can result in an inward Na^+^/Ca^+ ^exchange current (I_NCX_) that contributes to the diastolic depolarization rate and to the initiation of the AP. 

The SAN displays a functional and anatomic inhomogeneity because SAN cells have different intrinsic cycle lengths (CLs) or interbeat intervals (IBIs), which correspond to the interval between consecutive AP upstrokes. Moreover, SAN cells differ in terms of responsiveness to ions, drugs, temperature or neuro-hormones [28]. These differences are related with regional anatomic and molecular heterogeneity of single cells within the node [29,30]. For instance, cells from the center of the SAN are small and have a more positive resting membrane potential, a slower diastolic depolarization phase, and slower AP upstroke, compared to larger cells that are found in the periphery. Multiple ion channels contribute to the differences in AP morphology of central and peripheral SAN cells (for review, see [26, 29]). In addition, nodal cells in the center of the SAN are poorly electrically coupled due to low expression of connexins, which constitute the gap junctions. As a consequence, AP propagation in the center of the SAN is slow, making this region electrically insulated from the surrounding atrial muscle. However, expression of connexins Cx43 and Cx45 increases toward the periphery, thus improving electrical coupling [29,30]. 

This extensively distributed system of atrial pacemakers constitutes the pacemaker complex, which induces changes in heart rate and beat-to-beat cycle length due to frequent exchange of dominance between different pacemakers. Changes in any of the input signals may produce pacemaker shifts and alter prevailing beat-to-beat rate and variability. The same is true when dynamic competition between multiple pacemakers rather than multifocal origin of the impulse, or different areas of the leading pacemaker location site, produce changes in BRV [30].

### A Evidence of Beat-rate Variability: *In Vitro *Experiments

#### Beating Irregularity of Single Pacemaker Cells and Monolayer Cultures of Cardiac Cells

Jose and Collison [31] defined ‘inherent heart rate’ as the heart rate under the simultaneous presence of β adrenoreceptor antagonist propranolol and the muscarinic receptor blocker atropine. Rosenblueth and Simeone [32] proposed a formula that links inherent heart rate (HR_0_) with sympathovagal inputs upon sinoatrial node: HR = m*n* HR_0_, where the product m*n represents sympathetic and vagal tone, respectively. Despite its simplicity, the above relation demonstrates a continuous cross-talk between the two components of heart rate control, ANS and *in situ *SAN pacemaker activity. 

Pacemaker activity has been found to exhibit continuous variability of IBI (BRV) in different *in vitro *experimental studies. Clay and DeHaan [28] observed random fluctuations of CLs in spontaneously firing cells isolated from embryonic chick heart. They hypothesized that such fluctuations arise from inherent variability of diastolic depolarization towards AP threshold, due to stochastic open-close kinetics of membrane ionic channels. In addition, they found that SAN cells’ beating irregularity diminishes with increasing aggregate cell size, whereas the co-efficient of variation is approximately inversely proportional to the square root of the number of interconnected cells’ [28]. 

Jongsma, *et al*. [33] came to the same conclusion in terms of BRV in neonatal rat atrial and ventricular cardiac cells, with an IBI coefficient of variation around 2%. They found that IBI variability diminished when cells were interconnected by gap junction channels. Jongsma, *et al*. noted: ‘Whereas individual pacemaker cells discharge irregularly, denervated SAN exhibits a regular discharge pattern. The hypothesis of explanation is that small SAN cells contain a few channels that give rise to observed voltage fluctuations, whereas in the intact SAN the membrane of combined cells are connected in parallel by resistive coupling between the cells. Due to the huge increase in the total number of ionic channels, individual variations are averaged out and firing threshold is reached at the same time in every cycle’ [33]. Another possible influence upon BRV derives from surrounding atrial cells. Using mathematical models, Joyner and van Capelle [34] found that electrotonic interactions may play a significant role in beating rate irregularity of the intact SAN and Wilders and colleagues [35] revealed an increase in IBI coefficient of variation of around 2.9% induced by ‘atrial load’. In the latter study, it was also proposed that a gradation in coupling resistance from the center of SAN towards the atrium is necessary for impulse propagation [35]. 

Kucera *et al*. [18] studied BRV monolayers with spontaneously beating neonatal rat ventricular myocytes, devoid of extracardiac influences, using unipolar extracellular electrograms. The beat-rate time series were examined with Fast Fourier transformation and Hurst scaling exponents’ calculation, a mathematical method used for assessing fractal properties of different time series. In 21 from the 22 monolayers studied, the authors found a power law behavior with a β exponent around -1.35 [18], Typically, healthy subjects have a value lower than -1, while after myocardial infarction and after cardiac transplantation it is between -1.2 and -2 [16]. Lower or more negative β slope values correspond to time series with a closer resemblance to Brownian motion (for which β = -2) [11]. The findings of Kucera and colleagues support the hypothesis that ‘during cardiac diseases, a partial break-down of autonomic control may unmask components of HRV intrinsic to cardiac tissue’. In addition, Kucera *et al*. [18] found that β slope during long lasting experiments displays a high standard deviation, suggesting that autonomic modulation of BRV might stabilize β slope. 

Another study of Kucera and coworkers [19] demonstrated that both the movement of the beat-to-beat focus and variability exhibited power law behavior. Stochastic fluctuations in trans-membrane potential currents and gating of Ca^2+^ release channels were not responsible for such long term correlations. However, turnover of various ion channels reproduced fractal properties responsible for intrinsic beating variability [19]. 

Yokogawa and Harada [20] evaluated power law behavior of beat rate time series in isolated atrial and ventricular myocytes, in order to assess inherent fractal properties of cell membranes irrespective of cell network interactions that arise within a monolayer culture. Timing of spontaneous contractions was determined from a sequence of phase contrast microscopic images, based on changes in brightness of pixels. Using a method called detrended fluctuation analysis (DFA) for the assessment of autocorrelation of the contraction timings they observed similarity between atrial and ventricular myocytes in terms of long-term correlations of beat rate fluctuations. According to the authors [20], their findings describe a universality of such long-term properties of beat rate fluctuations, since they were found in cells from different chambers of the heart. In a closely related study [36], the same authors proposed that fluctuations in ATP concentrations, reflecting the metabolic state of the cell, might be the cause of long-range correlations characterizing BRV, through activation of ATP-regulated K^+^_P _ currents. However, they concluded that this mechanism is only valid for neonatal ventricular cells that exhibit spontaneous activity, and not for mature ventricular myocytes. SAN pacemaker cells have different characteristics, such as type of ionic current channels, signal transducing proteins etc, limiting generalization of results in different experimental settings [29]. 

In addition, Wilders and Jongsma [37] observed that in isolated SAN node cells, the auto-correlation function of IBIs drops to 0 immediately after a lag of 1 beat. This means that there are no long range correlations and that the power spectrum is flat. Thus, there is no power law behavior in the *in vitro* single cell model of Wilders and Jongsma, which is at odds with previous studies in which a power law behavior was observed [18-20]. It seems that differences between cells from different chambers of the heart or electrotonic interactions between interconnected cells might change their fractal properties, reflected in power-law behavior.

#### Pacemaker Cells’ Response to Autonomic Inputs 

SAN pacemaker activity can be viewed as a transduction mechanism between input signals, such as neural, endocrine and mechanical factors and output signal that is the cycle length of pacemaker discharge [26]. Rocchetti *et al*. [38] investigated the effects of muscarinic receptor activation upon CL variability in isolated SAN cells of rabbits using the patch clamp technique. In today’s cardiac cellular electrophysiology, patch-clamp, as described in detail by Kornreich [39], is the common technique to record the electrical activity of single cells [24,25]. 

Using the patch clamp technique, Rocchetti *et al*. [38] found that SAN cells exposed to different acetylcholine (Ach) concentrations displayed a significant short-term variability of CL, estimated with its standard deviation, which was increased exponentially in a dose-dependent fashion. This implies a non-linear relation between input/output signals at the level of SAN, where a constant level of receptor input can affect both the level and the variability of the output pacemaker discharge. According to Zaza and Lombardi [40], the reduction in CL variability in different pathologic states could reflect both a reduced input from ANS and a blunted response of the SAN to autonomic influences. Finally, any change in diastolic depolarization rate through alterations in different ionic currents of pacemaker cells might modulate CL variability. One possible explanation could be the shift of leading pacemaker between regions within the SAN with different receptor densities and sensitivity, associated with changes in neural activation, temperature or extracellular ionic concentrations. In addition, LF periodicities in the concentration of second messenger substances that can affect BRV could be attributed to the existence of mutually antagonistic signaling systems (e.g kinases versus phosphatases), which are altered by neural and chemical inputs [40]. 

### B Evidence of Beat-rate Variability: *Ex Vivo *Experiments

#### From Cellular Dynamics to Whole Heart Preparations

There are a limited number of studies in the literature investigating origins of HRV in isolated hearts. In 1999, Langer and colleagues [21] were the first to quantify the effects of afterload, preload, and temperature changes on IBI variability in intact isolated hearts of Sprague-Dawley rats. Using C _90_, the central 90% range of the beating intervals during 10 mins periods, as a time domain method of assessment of HRV and frequency domain methods, they found that changes in end-diastolic and aortic pressures had no effect, where decrease in temperature from 37 °C down to 27 °C increased C _90 _about sevenfold. The beating intervals of isolated SAN cells were found to be normally distributed with significant variability. On the contrary, isolated rabbit right atrium and more pronouncedly, the intact rat hearts exhibited significantly smaller IBI fluctuations [21]. According to Langer and colleagues [21], at low heart rates, as typically occur during hypothermia, the steepness of the diastolic depolarization is reduced, leading to a variation of the trigger point that corresponds to the onset of AP.

The SAN includes clusters of pacemaker cells depolarizing independently with a multi-centric origin of a depolarization wave [29]. Since SAN cells exhibit the phenomenon of mutual synchronization, simulation studies from Michaels [41] demonstrated that a reduction in gap junction densities and conductivity may cause a decrease in intercellular connections, leading to a more prolonged and heterogeneous propagation of mutual synchronization. This decrease in the effective size of pacemaker cell clusters is responsible for the increase in IBI variability, estimated with C_90_, during hypothermia. In addition, individual BRV could be associated with different size of pacemaker clusters, implying that every factor that diminishes inter-node conductivity, such as low temperature, or accelerates conduction velocity, such as catecholamine application, can produce an increase or decrease in BRV, respectively [21]. Another finding from the study of Langer was that the SAN is the sole source of IBI variability, since neither preload or afterload conditions nor impulse conduction variation, which has been associated with the fractal structure of His-Purkinje network by Goldberger [42], contributed to the observed CLs variations.

More recently, Frey *et al.* [43] analyzed HRV in adult rabbit hearts, perfused for twenty minutes in a Langendorff set-up. An inherent HRV was found using metrics from the frequency domain (total power as an index of overall variability) in isolated denervated hearts, whereas both left atrial and ventricular chamber filling did not affect HRV. In another experimental study, Janousek *et al. *[44] compared HRV in *ex vivo *and *in vivo *whole heart preparations of rabbits and found a significant decrease in all frequency domain metrics of HRV in the first, compared to the second group. In addition, LF/HF ratio was five times higher in isolated heart than in *in vivo *hearts. 

Monfredi and colleagues [abstract] tried to unmask possible mechanisms of HRV in isolated Langendorff perfused hearts of rabbits. They found that HRV, estimated with SDNN and power spectrum density, existed in denervated cardiac preparations and was significantly higher in isolated pacemaker cells than in whole hearts. Lack of effect of both atropine and propranolol that were infused in isolated hearts suggests the presence of HRV unrelated with possible residual effect of inherent autonomic ganglia. Moreover, HRV in terms of total power was decreased after administration of drugs which affects intracellular Ca^2+^ homeostasis, such as ryanodine, implying that Ca^2+^ release from the SR could be a possible mechanism of HRV generation. Finally, application of Cs^+^, frequently used in experimental laboratories as I_f_ inhibitor [24,25], increased total power of heart rate signals. 

Table **1** summarizes major findings from studies discussed so far, regarding the presence of inherent beat-rate variability with power law behavior in different experimental *in vitro *and *ex vivo *studies.

## HRV AND FUNNY CURRENT I_F_ IN CARDIOVASCULAR AND CRITICAL CARE MEDICINE

### Clinical Implications of Low HRV 

The first large prospective population study that proved the significant prognostic value of low HRV after an acute myocardial infarction was the Autonomic Tone and Reflexes After Myocardial Infarction Study (ATRAMI), and included 1284 patients with a recent (<28 days) myocardial infarction [4]. A 24 h Holter recording was done to quantify HRV (using SDNN values) and ventricular arrhythmias. Low values of HRV (SDNN<70 ms) carried a significant multivariate risk of cardiac mortality. Furthermore, the association of low SDNN with left ventricular ejection fraction <35% carried a relative risk of 6.7, compared with patients with LVEF >35%. In the Framingham Heart Study [45], HRV time (SDNN) and frequency domain measures were computed in 736 patients and correlated with all-cause mortality, during 4 years of follow-up. The authors concluded that HRV offers prognostic information for mortality independent of that provided by traditional risk factors. 

In the Zutphen study [46], 885 middle-aged (40-60 years old) and elderly Dutch men (aged 65-85) were followed from 1960 until 1990, whereas SDNN was determined from the resting 12-lead ECG. It was shown that low HRV is predictive of mortality from all causes, indicating that it can be used as an index of compromised health in the general population. It seems that the predictive value of low SDNN is independent of other factors, such as depressed left ventricular ejection fraction and presence of late potentials. 

It is supposed that the change in the geometry of a beating heart due to necrosis may abnormally increase the firing of sympathetic afferent fibers by mechanical distortion of their sensory endings [47,48]. This excitation attenuates the vagal activity to the SAN. After acute myocardial infarction, HRV is reduced for a period of few weeks, while it is maximally, but not fully, recovered after 6 to 12 months [49].

In addition, retrospective ECG data analysis from 127 patients included in the Veterans Affairs’ Survival Trial of Antiarrhythmic Therapy in Congestive Heart Failure (CHF) [50] demonstrated that CHF patients with SDNN <65.3 ms had a significantly increased risk of sudden death. Moreover, this study demonstrated that every 10 ms increase in SDNN conferred a 20% decrease in risk of mortality. 

Furthermore, it has been demonstrated that subclinical inflammation and the concentration of inflammatory markers, such as cytokines, correlate strongly with cardiovascular mortality and morbidity in both healthy subjects and in those with known coronary artery disease (CAD) [51,52]. In this respect, a limited number of clinical studies investigating a possible association between ANS outflow and various inflammatory indices in patients with heart diseases have appeared in the literature [53-55]. 

Lanza and colleagues [53] assessed HRV and measured C-reactive protein (CRP) serum levels within 24 hours of admission in 531 patients with unstable angina pectoris. They found a small but statistically significant negative correlation between CRP levels and all HRV metrics derived from both time and frequency analysis, with the highest r coefficient with SDNN and VLF. After categorizing patients into 4 subgroups according to CRP quartile levels, significantly lower HRV (low SDNN) values were found in the upper CRP quartile. The subsequent multivariate analysis revealed that SDNN and VLF were the most significant predictors of increasing CRP, whereas CRP itself was proven to be a strong predictor of impaired ANS activity as well. Psychari *et al.* [54] also reported a strong inverse association between CRP and several HRV indices (SDNN, HF and LF) in patients after acute myocardial infarction after adjustment for left ventricular function. 

Malave *et al.* [55] examined HRV in relation to circulating levels of tumor necrosis factor-α (TNF-α), TNF receptors and norepinephrine in 10 controls, 15 patients with mild CHF and 14 subjects with moderate heart failure. They observed a significant inverse linear correlation between increased levels of all biomarkers and SDNN, LF and HF power among CHF patients [50]. In addition, the LF power was more closely correlated with circulating levels of TNF-α than was the HF component, whereas multiple linear regression analysis showed that TNF-α was a stronger predictor of reduced HRV than was the circulating levels of norepinephrine [55]. Therefore it was concluded that over-expression of TNF-α and subsequent loss of β-adrenergic responsiveness contributes to the decrease in HRV observed in heart failure. According to recent experimental studies, TNF-α might inhibit β-adrenergic signal transduction through either activation of Gi proteins or impairment of activation of Gs proteins [56], something that could be viewed as an adaptive mechanism in the early stages of CHF through protection of cardiac myocytes from the deleterious actions of catecholamines. However, in the more advanced stages of the disease, this mechanism could become maladaptive, leading to a reduction in cardiac output [57]. 

Similar results in terms of alterations in HRV during septic shock and multiple organ dysfunction syndrome have been reported from different research groups [2,3,5]. For instance, using spectral analysis of HRV and blood pressure of critically ill patients, Goldstein *et al.* [5] was able to show that increased total variability and LF power were associated with recovery and survival, whereas a decrease in total power, LF/HF and LF power correlated with severity of illness and mortality in septic patients, 48 hours after being admitted to the Intensive Care Unit (ICU). This loss of variability of heart rate signals has been attributed to a ’decomplexification’, which is associated with a defective communication between different organs, due to ANS dysfunction and parallels severity of disease [5,58]. Moreover, early HRV alterations could reflect different cytokine patterns as their local production can be significantly increased by denervation of an organ [59]. In this respect, Tateishi *et al.* [60] investigated the relationships between HRV and interleukin (IL) 6 upon admission in a cohort of 45 septic patients and they found that IL6 exhibited significant negative correlations with both LF and HF power values. These finding indicate an association between low HRV indices and hyper-cytokinemia, suggesting that there is a continuous cross-talk between ANS and immune-regulated inflammatory response.

### HRV and I_f_ in Critical Illness: Lessons from Cardiac Cellular Electrophysiology

The reasons for reduced HRV, estimated with time or frequency domain methods, which has been found in different pathologic states, such as myocardial infarction [4] and severe sepsis or septic shock [5], have been debated and two theories have been developed. The first theory focuses on reduction of vagal tone and has been introduced by Akselrod *et al.* [9]. The second theory developed by Goldberger and colleagues [61] states that normal physiology has fractal-like properties with high levels of complexity that explain phenomena such as HRV. Severe disease reflects a ‘decomplexification’ mostly attributed to uncoupling between different restorative mechanisms [58]. Accumulating evidence from both *in vitro *and *ex vivo *experiments and human studies, with cardiac transplant recipients with hearts devoid from autonomic nervous inputs, support a potential third mechanism proposed recently by Griffin *et al.* [62], which is associated with an intracardiac origin of HRV. According to this hypothesis, SAN cells with an extreme heterogeneity in electrophysiological properties and intercellular connections of SAN cells can be viewed as an amplifier of various input signals [40]. During severe sepsis or in cardiovascular diseases, an unfavorable metabolic milieu could affect ion channel gating properties or membrane receptor densities, with significant impact upon level and variability of pacemaker activity. In addition, a possible reduced responsiveness of SAN cells to external stimuli could also negatively affect HRV [62]. 

In this respect, Janousek and colleagues [63] studied *ex vivo *heart preparations from rabbits, and using different protocols of ischemia and reperfusion, they computed thirty-five indices of HRV. They concluded that ischemia decreases significantly two metrics that seem to reflect intracardiac mechanisms of HRV, the high frequency component and SD2, which is the standard deviation of the Poincaré plot (RR n+1 versus RRn) along the line of the identity. The last method is used as a visual tool for variability assessment in different time-series. In a study of isolated SAN cells from control rabbits and rabbits with volume and pressure overload-induced heart failure, Verkerk *et al*. [64] found increased intrinsic CL and decreased diastolic depolarization rate, associated with a reduced density of I_f_. This is supported by findings in dogs, where heart failure results in down regulation of HCN4 and HCN2 expression in the SAN [29].

Administration of lipopolysacchraride (LPS, endotoxin derived from the cell wall of Gram-negative bacteria) has been found to decrease HRV [5,15]. In an animal study of experimental endotoxemia, Fairchild *et al. *[65] demonstrated a strong inverse correlation between SDNN and total power of RR time series and peak concentrations of different cytokines, 3-9 hours post-LPS. The same results were found after administration of recombinant TNF-α. Mechanisms responsible for decrease in HRV could be related with effects of LPS and/or cytokines on various ion channels.

Zorn-Pauly and colleagues [66] studied the effects of LPS on I_f_ in human myocytes isolated from atrial appendages of patients undergoing open-heart surgery. After incubation with 10 μgr/mL of LPS, for 6 to 10 hours they found that the voltage-dependency of I_f_ activation was shifted to more negative potentials. Consequently, I_f_ was significantly lower in the physiological voltage range for SAN pacemaker activity. Using a mathematical computer model of a SAN cell, the effects of LPS on I_f_ resulted in a longer CL. Using the mathematical SAN model cells, they further showed a reduced response of IBI fluctuations to ANS stimuli after LPS-induced I_f _impairment. A reduced responsiveness to external autonomic inputs may affect HRV. According to the authors ‘altered I_f _gating during endotoxemia may originate from conformational changes, induced by direct binding of LPS to the channel and/or changes in membrane properties’ [66]. The results of Zorn-Pauly *et al.* agree with the findings of Khaykin and colleagues [67], who showed that 10 mg of zatebradine, an I_f _blocker, reduced all frequency components of HRV in patients without structural heart disease. In addition, Joannides *et al. *[68] found that Ivabradien, a new selective I_f_ current blocker at the level of SAN, significantly decreased LF/HF ratio in healthy volunteers during tilt and exercise. 

However, in another experimental study [69], an increased HRV estimated with SDNN after administration of zatebradine was observed in conscious rats, three days after coronary artery ligation or sham-operation. Such results could support the hypothesis of Noble and DiFransesco [70] regarding the stabilizing effect of I_f_ upon pacemaker frequency, ‘against changes induced by altering other conductances *whatever the magnitude of its contribution to the depolarizing current’*. Reasons for such discrepancies could be differences in study design (early versus late administration, amount of dose, pre-existing pathology, or differences between species). In addition, I_f_ blockers can affect HRV in different ways, either through a modulation of heart rate, effects upon baroreflex sensitivity in *in vivo *experimental settings through central action [71] or depending on levels of stress [69].

Recently, the BEAUTI_f_UL trial (MorBidity-mortality EvalUation of the I_f _inhibitor Ivabradien in patients with coronary disease and left ventricUlar dysfunction) including 10.917 patients with CAD, LVEF < 40% and heart rate ≥ 60 beats/min [72], has shown that Ivabradien in patients with a heart rate of >70 beats/min decreased admission to the hospital for fatal and non-fatal myocardial infarction or coronary revascularization [73]. Moreover, the SHIFT trial (Systolic heart failure treatment with I_f _inhibitor Ivabradien trial) including patients with stable symptomatic heart failure, LVEF ≤ 35% and heart rate ≥ 70 beats/min, showed a significant reduction in deaths from heart failure after heart rate reduction with Ivabradien [74]. 

Effects of LPS upon heart rate in septic patients seem to involve rather inherent heart rate than ANS influences at the level of SAN [75]. In different studies it has been shown that tachycardia in sepsis develops slower and lasts longer compared to reduction in blood pressure, or even continues after adequate volume resuscitation. In addition, septic tachycardia is apparent even after a decline in the plasma levels of catecholamines, indicating a no baroreceptor mediated mechanism [76,77]. Finally, sepsis seems to increase sympathetic tone at the level of peripheral nerve endings independent of increases in firing heart rate [78].For these reasons, it has been suggested that septic tachycardia is due to an increase in *in vitro *inherent heart rate [79]. 

This non-autonomic origin of increased heart rate in sepsis is paradoxically associated with desensitization of beta-receptors [80], whereas analysis of HRV in septic animals by Goldstein *et al*. [81] has found a decrease or absence of sympathetic input upon the heart, estimated with LF/HF and LFnu, despite a decrease in overall HRV. However, these experiments do not elucidate whether loss of HRV is related to an endotoxin effect at the level of ANS output, baroreflex sensitivity or the pacemaker cell itself [79]. In an experimental study of single intraperitoneal LPS administration of 5 mg/Kg in rats, Wearden [82] showed that inherent heart rate increased at approximately 4 hours after endotoxin injection, whereas LFnu and LF/HF ratio did not change significantly. In addition, total power of HRV began to decrease at 2 hours, reaching its minimal values 7 hours later. According to Wearden [82], loss of HRV estimated with total power of frequency spectrum, reflects baroreceptor mediated mechanisms of heart rate variability, whereas increased inherent heart rate might be due to an unknown cellular mechanism. Moreover, this mechanism could initiate an LPS-induced septic tachycardia, however, catecholamines are required to maintain this effect. The fact that tachycardia in the absence of significant sympathetic input upon the heart, as it was estimated with HRV analysis, paralleled a decrease in contractility, could reflect a maladaptative mechanism responsible for an increase of oxygen delivery to the tissues with less autonomic stimulation [82]. 

Time changes of HRV and inherent heart rate during sepsis might reflect study designs. In different *in vivo *experimental septic models that include different doses or routes of administration of LPS (i.e., intravenous bolus, cecal ligation or puncture models), it has been shown that there is a varying time lag after injection of endotoxin before endotoxemia develops [83]. This may be due to different time of peaks in the concentration of various cytokines [84]. However, different experimental conditions, i.e., animal’s weight, temperature, anesthetic agents, residual effects of catecholamines or even doses of LPS can bias such findings significantly [86-87]. 

The finding that I_f_ could also mediate HRV changes during endotoxemia [66], might explain findings from other studies concerning both septic tachycardia and loss of overall HRV [75,81]. Although, the combination of a markedly attenuated vagal tone that is found in severe sepsis [79], a massive release of endogenous catecholamines or their exogenous administration could override* in vivo* the bradycardic effects of LPS*,* endotoxin can also sensitize the HCN channels for sympathetic stimulation, thereby increasing heart rate [66]. As a consequence, a combination of increased heart rate and narrowed HRV indicate autonomic dysfunction and poorer prognosis in patients with multiple organ dysfunction syndrome , who display similar findings in terms of pathophysiologic mechanisms, with those suffering from chronic heart failure [88]. 

The above association is based on the presence of sympathetic overactivity, autonomic dysfunction, inappropriately increased heart rate, insulin resistance and in some cases, cardiomyopathy with reduced cardiac contractility [88,89]. As with heart failure, a down regulation of beta receptors is also found in septic patients, limiting the possible use of beta blockers [80]. In addition, increased heart rate in these patients has been associated with fatal outcome [90]. However, in cardiac patients sympathetic activity dominates over vagal tone where in MODS patients both branches of ANS are attenuated [79]. For these reasons, it has been hypothesized that during critical illness except for ANS impairment, a defective signal transduction at the level of pacemaker cells could also account for observed differences between cardiac and septic patients. I_f_ could be a possible candidate due to its reduced amplitudes during endotoxemia. In this respect, a possible beneficial effect of I_f_ inhibitors merits further investigation and could shed more light into the role of I_f_ in septic cardiomyopathy. A randomized, controlled phase-2 trial –the MOD*I*_f_ *Y* trial- is underway (www. clinicaltrials. gov, NCT 01186783), trying to explore a possible benefit from the use of Ivabradien in 70 critically ill patients with multiple organ dysfunction syndrome, with heart rate ≥ 90 beat/min and contraindication to beta-blockers [91]. 

There are a few more studies in the literature, trying to investigate a possible impact of LPS upon HRV, at the cellular level. In monolayers of spontaneously contracting rat neonatal ventricular cardiomyocytes, Schmidt and colleagues [92] showed that application of 1 μgr/mL of LPS significantly reduced SDNN, RMSSD and pNN50. Since the authors did not study real SAN cells, they hypothesized that endotoxemia induces an ionic remodeling, based on already proven negative effects of LPS upon different ion channels, such as a decrease in density and activation properties of L-type Ca^2+^ channels and down regulation of β-adrenergic receptors [79,89]. 

Recently, Wondergem *et al. *[93] confirmed the previous results in immortalized HL-I cardiomyocytes. Application of 1 μgr/mL of LPS resulted in a rapid reduction in Ca^2+^ oscillations and total intracellular Ca^2+^ concentration. In addition, I_F _was inhibited at very negative potentials. The effects occurred rapidly after LPS application (within minutes), which argue against the contribution of release of second messengers, such as cytokines and cAMP. 

One more study that was performed in Human Embryonic Kidney 293 (HEK293) cell lines, confirmed negative effects of LPS on I_f _properties. Klöckner *et al* [94] transfected HEK cells with cDNA encoding the HCN2 channel proteins, for comparison reasons with Zorn-Pauly findings [66], since these HCN transcripts are predominantly expressed in human right atrium myocytes [95]. LPS was found to induce NF-kB activation through Toll-like-receptors-4 (TLR-4), leading to IL-6 production within 1-2 hours that reached its maximum after more than 24 hours. However, decrease in activation of I_f _with reduced steepness of diastolic depolarization seemed to occur within approximately 8 seconds. As a result, it was shown that the polysaccharide part of LPS ‘O-chain’ is required for immediate reduction of HCN channel activity. Thus, endotoxin seems to affect with different structures of its molecule the activation of the host immune system and the modification of HCN channel electrophysiological properties. According to Klöckner *et al* [94], their results parallel reports from the anesthesia literature, where both propofol and halothane induced a dose-dependent inhibition of HCN channel activity and an associated decrease in heart rate variability [96,97]. However, possible differences of biophysical properties of various HCN transcripts could bias previous results, since these isoforms have been found to co-assemble in varying ratios, thus modulating SAN responsiveness to different stimuli [98]. 

Table **2** presents some of the studies investigating the role of funny current in cardiovascular diseases and sepsis, in both experimental and clinical settings. 

## CONCLUSIONS AND FUTURE SUGGESTIONS

Contradictory results in terms of HRV changes during severe sepsis and MODS have been found, mainly due to differences in experimental design, (i.e. differences in species studied, isolated SAN cells versus intact SAN preparations and/or intact hearts, etc). In addition, sepsis and MODS are considered extremely complex and available experimental models fail to represent accurately human diseases. Although in cardiovascular medicine different experimental and clinical studies have managed to confirm the value of HRV measurement in the clinical setting, critical illness still remains an open field for future investigation, concerning implementation of HRV quantitative analysis and evaluation of its potential for risk stratification or even building different prognostic models. In addition, understanding of the complex mechanisms responsible for its generation requires *in vitro *or *ex vivo *models, representative of different diseases, in order to develop future therapies for cardiovascular and septic patients, targeting increased heart rate. 

Existing literature supports the presence of intracardiac mechanisms of heart rate variability at the level of either membrane channel kinetics or intracellular transduction signaling. However, and despite evidence from numerous studies that demonstrate negative inotropic effects of LPS during sepsis [99], there are only a limited number of reports investigating its chronotropic effects, particularly in association with inherent heart rate and loss of beat-to-beat rate variability in animal septic models. 

The complexity of HRV and the existing debate regarding its origin puts another obstacle for understanding underlying pathophysiology and assessing its value as a monitoring tool, in terms of both prognosis and treatment effect, in different groups of patients. Many details remain to be discovered, such as effects of LPS on conductivity between SAN pacemaker cells, since recent findings support a reduced expression of various gap junction proteins in ventricular cells during severe sepsis [100]. In addition, association of sepsis with pacemaker shifts, ionic remodeling of different currents responsible for spontaneous diastolic depolarization or membrane potential propagation within the SAN, could be some interesting areas for future research, in relation with reduced HRV as an index of cellular metabolic stress. Moreover, the use of simulation studies with mathematical models that are more valid for larger mammal’s SAN electrophysiological properties might be of significant value in the near future. 

Finally, standardization of experimental protocols and methods used for the estimation of HRV and the use of open-source data and knowledge bases, such as the website physionet (www. physionet.org) [6], with different analytic tools that can be adopted for free by independent investigators, is an ongoing effort that could enhance understanding and implementation of this technology in both basic and clinical research. 

## Figures and Tables

**Table 1.** Summary of Different Experimental *In Vitro* and *Ex Vivo* Studies Concerning the Presence of Beat-rate Variability (BRV) and Power-law Behavior of Isolated Cells and Denervated Hearts

Protocol	Authors	Methods	Findings
Spontaneously firing cells isolated from embryonic chick hearts	Clay and DeHaan [28]	Coefficient of variation (CV)	CV was inversely proportional to the square root of the number of interconnected cells
Neonatal rat atrial and ventricular cardiac cells	Jongsma, *et al*. [33]	Coefficient of variation (CV)	CVs diminished when cells were interconnected by gap junction channels
Monolayers with spontaneously beating neonatal rat ventricular myocytes	Kucera *et al*. [18]	Fast Fourier transformation (FFT) and power law β exponent	Power law behavior with a β exponent around -1.35
Isolated atrial and ventricular myocytes	Yokogawa and Harada [20]	Detrended fluctuation analysis (DFA) estimating fractal properties	Similarity between atrial and ventricular myocytes in terms of long-term correlations of beat rate fluctuations
Effects of afterload, preload, and temperature changes on IBI variability of intact isolated hearts of Sprague-Dawley rats	Langer and colleagues [21]	C _90 _as a measure of CV and FFT	Isolated rabbit right atrium and rat hearts exhibited significantly smaller IBI fluctuations related with isolated SAN cells
Adult rabbit hearts	Frey *et al.* [43]	FFT (total power)	Presence of inherent HRV in isolated hearts

**Table 2.** Summary of Different Experimental and Clinical Studies Concerning the Role of Funny Current During Sepsis and Heart Failure

Authors	Experimental / Clinical protocol	Major findings
Verkerk *et al*. [64]	SAN cells from control rabbits versus rabbits with volume and pressure overload-induced heart failure	Increased intrinsic CL and decreased diastolic depolarization rate, associated with a reduced density of I_f_.
Zorn-Pauly and colleagues [66]	Effects of LPS on I_f_ in human myocytes isolated from atrial appendages of patients undergoing open-heart surgery.	Voltage-dependency of I_f_ activation was shifted to more negative potentials. Reduced response of IBI fluctuations to ANS stimuli after LPS-induced I_f _impairment
Joannides *et al. *[68]	Healthy volunteers during tilt and exercise.	Ivabradien, a new selective I_f_ current blocker at the level of SAN, significantly decreased LF/HF ratio
BEAUTI_f_UL trial [72,73]	10.917 patients with CAD, LVEF < 40% and heart rate ≥ 60 beats/min	Ivabradien in patients with a heart rate of >70 beats/min decreased admission to the hospital for fatal and non-fatal myocardial infarction
MOD*I*_f_ *Y* trial [91]	Effects of Ivabradien in 70 critically ill patients with multiple organ dysfunction syndrome, with heart rate ≥ 90 beat/min and contradiction to beta-blockers	(still ongoing)
Schmidt and colleagues [92]	Impact of LPS upon HRV (SDNN, RMSSD), in monolayers of spontaneously contracting rat neonatal ventricular cardiomyocytes	1 µgr/mL of LPS significantly reduced SDNN, RMSSD and pNN50
Klöckner *et al* [94]	Transfected HEK cells with cDNA encoding the HCN2 channel proteins and incubated with LPS	Decrease in activation of I_f _with reduced steepness of diastolic depolarization, occured within approximately 8 seconds

## ABBREVIATIONS

AP: = action potential
ANS: = autonomic nervous system
BEAUTIFUL: = MorBidity-mortality EvalUation of the I_f_ inhibitor ivabradien in patients with coronary disease and left ventricUlar dysfunction
BRV: = beating rate variability
CL: = cycle length
CAD: = coronary artery disease
CHF: = congestive heart failure
CRP: = C-reactive protein
DFA: = detrended fluctuation analysis
ECG: = electrocardiogram
FFT: = Fast Fourier transformation
HRV: = heart rate variability
HR_0_: = inherent heart rate
HF & HFnu: = high frequency and normalized HF values
HEK: = human embryonic kidney cells
HCN: = Hyperpolarization-activated Cyclic Nucleotide
IBI: = inter-beat intervals
ICU: = intensive care unit
LF & LFnu: = low frequency and normalized LF units
LVEF: = left ventricular ejection fraction
LPS: = lipopolysacchraride
MODS: = multiple organ dysfunction syndrome
pNN50: = proportion derived from dividing NN50 (number of interval differences of successive intervals greater than 50 ms) by the total NN intervals
PSD: = power spectrum density
RMSSD: = square root of the mean squared differences of successive intervals
RAS: = rennin-angiotensin-aldosterone
RSA: = respiratory sinus arrhythmia
SAN: = sinus atrial node
SDNN: = standard deviation of the normal-to-normal intervals which is the square root of the variance
SHIFT: = Systolic heart failure treatment with I_f_ inhibitor ivabradien trial
SR: = sarcoplasmic reticulum
SD: = standard deviation
TLR: = Toll-like receptor
VLF: = very low frequency

## References

[1] Buchman TG (2002). The community of the self. Nature.

[2] Seely AJE, Christou NV (2000). Multiple organ dysfunction syndrome: exploring the paradigm of complex nonlinear systems. Crit Care Med.

[3] Goldstein B, Buchman TG (1998). Heart rate variability in intensive care. Intensive Care Med.

[4] La Rovere MT, Bigger JT, Marcus FI, Mortara A, Maestri R, Schwartz PJ (1998). Baroreflex sensitivity and heart rate variability in prediction
of total cardiac mortality after myocardial infarction.
ATRAMI (Autonomic Tone and Reflexes After Myocardial Infarction)
Investigators. Lancet.

[5] Goldstein B, Fiser DH, Kelly MM, Mickelsen D, Ruttiman U, Pollack MM (1998). Decomplexification in critical illness and injury: Relationship between heart rate variability, severity of illness, and outcome. Crit Care Med.

[6] Goldberger AL, Amaral LAN, Glass L (2000). PhysioBank, PhysioToolkit, and PhysioNet: Components of a new research resource for complex physiologic signals. Circulation.

[7] Priori SG, Aliot E, Blomstrom-Lundqvist C (2001). Task Force on Sudden Cardiac Death of the European Society of Cardiology. Eur Heart J.

[8] (1996). Task Force of the European Society of Cardiology and the North
American Society of Pacing and Electrophysiology. Standards of
measurement, physiological interpretation and clinical use. Circulation.

[9] Akselrod S, Gordon D, Ubel FA, Shannon DC, Barger AC, Cohen RJ (1981). Power spectrum analysis of heart rate fluctuation: a quantitative probe of beat to beat cardiovascular control. Science.

[10] Malik M, Camm AJ (1993). Components of heart rate variability: What they really mean and what we really measure. Am J Cardiol.

[11] Bigger JT, Steinman RC, Rolnitzky LM, Fleiss JL, Albrecht P, Cohen RJ (1996). Power law behavior of RR-interval variability in healthy middle-aged persons, patients with recent acute myocardial infarction and patients with heart transplants. Circulation.

[12] Montano N, Gnecchi-Ruscone T, Porta A (1996). Presence of vasomotor and respiratory rhythms in the discharge of single medullary neurons involved in the regulation of cardiovascular system. J Auton Nerv Syst.

[13] Lanfranchi PA, Somers VK (2002). Arterial baroreflex function and cardiovascular variability: inter-actions and implications. Am J Physiol Regul Integr Comp Physiol.

[14] DeBoer RW, Karemaker JM, Strackee J (1987). Hemodynamic fluctuations and baroreflex sensitivity in humans: a beat-to-beat model. Am J Physiol.

[15] Seeley A, Macklem P (2004). Complex systems and the technology of variability analysis. Critical Care.

[16] Goldberger AL (1996). Non-linear dynamics for clinicians: chaos theory.
Fractals and complexity at the bedside. Lancet.

[17] Thamrin C, Stern G, Frey U (2010). Fractals for physicians. Ped Respir Review.

[18] Kucera JP, Heuschkel MO, Renaud P, Rohr S (2000). Power-law behavior of beat-rate variability in monolayer cultures of neonatal rat ventricular myocytes. Circ Res.

[19] Ponard JGC, Kondratyev AA, Kucera JP (2007). Mechanisms of intrinsic beating variability in cardiac cell cultures and model pacemaker networks. Biophys J.

[20] Yokogawa T, Harada T (2009). Generality of a power-law long-term correlation in beat timings of single cardiac cells. Biochem. Biophys. Res. Commun.

[21] Langer SFJ, Lambert M, Langhorst P, Schmidt HD (1999). Interbeat interval variability in isolated working rat hearts at various dynamic conditions and temperatures. Res Exp Med.

[22] Bernardi L, Salvussi F, Suardi R (1990). Evidence for an intrinsic mechanism regulating heart rate variability in the transplanted and the intact heart during submaximal dynamic exercises. Cardiovasc Res.

[23] Hrushesky WHM, Fader D, Schmitt O (1984). The respiratory sinus arrhythmia: a measure of cardiac age. Science.

[24] Verkerk AO, van Ginneken AC, Wilders R (2009). Pacemaker activity of the human sinoatrial node: role of the hyperpolarization-activated current, I(f). Int J Cardiol.

[25] Verkerk AO, Wilders R, van Borren MM (2007). Pacemaker current (I(f)) in the human sinoatrial node. Eur Heart J.

[26] Mangoni ME, Nargeot J (2008). Genesis and regulation of the heart automaticity. Physiol Rev.

[27] Lakatta EG, Vinogradova TM, Maltsev VA (2008). The missing link in the mystery of normal auto-maticity of cardiac pacemaker cells. Ann N.Y Acad Sci.

[28] Clay JR, DeHaan RL (1979). Fluctuations in interbeat interval in rhythmic heart-cell clusters. Biophys J.

[29] Boyett MR, Honjo H, Kodama I (2000). The sinoatrial node: a heterogeneous pacemaker structure. Cardiovasc Res.

[30] Irisawa H, Brown HF, Giles W (1993). Cardiac pacemaking in the sinoatrial node. Physiol Rev.

[31] Jose AD, Collison D (1970). The normal range and determinants of the intrinsic heart rate in man. Cardiovasc Res.

[32] Rosenblueth A, Simeone FA (1934). The interrelations of vagal and accelerator effects on the cardiac rate. Am J Physiol.

[33] Jongsma HJ, Tsjernina L, DeBruijne J (1983). The establishment of regular
beating in populations of pacemaker heart cells. A study with
tissue-cultured rat heart cells. J Mol Cell Cardiol.

[34] Joyner RW, van Capelle FJL (1986). Propagation through electrically coupled cells: how a small SA node drives a large atrium. Biophys J.

[35] Wilders R, Jongsma HJ, van Ginneken ACG (1991). Pacemaker activity of the rabbit sinoatrial node: a comparison of mathematical models. Biophys J.

[36] Harada T, Yokogawa T, Miyaguchi T, Kori H (2009). Singular behavior of slow dynamics of single excitable cells. Biophys J.

[37] Wilders R, Jongsma HJ (1993). Beating irregularity of single pacemaker cells isolated from the rabbit sinoatrial node. Biophys J.

[38] Rochetti M, Malfatto G, Lombardi F, Zaza A (2000). Role of the input/output relation of sinoatrial myocytes in cholinergic modulation of heart rate variability. J Cardiovasc Electrophysiol.

[39] Kornreich BGF (2007). The patch clamp technique: Principles and technical considerations. J Vet. Cardiol.

[40] Zaza A, Lombardi F (2001). Autonomic indexes based on the analysis of heart rate variability: a view from the sinus node. Cardiovsc Res.

[41] Michaels DC, Matays EP, Jalife J (1987). Mechanisms of sinoatrial pacemaker synchronization: a new hypothesis. Circ Res.

[42] Goldberger AL, Bhargava V, West BJ (1985). On a mechanism of
cardiac electrical stability. The fractal hypothesis. Biophys J.

[43] Frey B, Hager G, Mayer C, Kiegler B, Stohr H, Steurer G (1996). Heart rate variability in isolated rabbit hearts. PACE.

[44] Janousek O, Ronzhina M, Sheer P, Novakova M, Provaznik I, Kolarova J (2010). HRV in isolated rabbit hearts and *in vivo* rabbit hearts. Comput Cardiol.

[45] Tsuzi H, Venditti FJ, Manders ES (1994). Reduced heart rate variability and mortality risk in an elderly cohort: the Framingham Heart Study. Circulation.

[46] Dekker JM, Schouten EG, Klootwijk P, Pool J, Swenne CA, Kromhout D from the ZUTPHEN STUDY (1997). Heart rate variability
from short electrocardiographic recordings predicts mortality from
all causes in middle-aged and elderly men. Am J Epidemiol.

[47] Kleiger RE, Miller JP, Bigger JT (1987). Decreased heart rate variability and its association with increased mortality after acute myocardial infarction. Am J Cardiol.

[48] Lombardi F, Sandrone G, Pernptuner S (1987). Heart rate variability as an index of sympathovagal interaction after myocardial infarction. Am J Cardiol.

[49] Bigger JT, Kleiger RE, Fleiss JL, Rolnitzki LM and the Multicenter
Post-Infarction Research Group (1998). Components of heart rate variability
measured during healing of acute myocardial infarction. Am J Cardiol.

[50] Bilchick KC, Fetics B, Djoukeng R (2002). Prognostic value of heart rate variability in chronic congestive heart failure (Veterans Affairs’ Survival Trial of Antiarrhythmic Therapy in Congestive Heart Failure). Am J Cardiol.

[51] Phillips AN, Neaton JD, Cook DG (1992). Leukocyte count and risk of major coronary heart disease events. Am J Epidemiol.

[52] Ridker PM, Cushman M, Stampfer MJ (1997). Inflammation, aspirin and the risk of cardiovascular disease in apparently healthy men. N Engl J Med.

[53] Lanza GA, Sgueglia GA, Cianflone D (2006). Relation of heart rate variability to serum levels of C-reactive protein in patients with unstable angina pectoris. Am J Cardiol.

[54] Psychari SN, Apostolou TS, Iliodromitis EK (2007). Inverse relation of C-reactive protein levels to heart rate variability in patients after acute myocardial infarction. Hellenic J Cardiol.

[55] Malave HA, Taylor AA, Nattama J, Deswal A, Mann DL (2003). Circulating levels of tumor necrosis factor correlate with indexes of depressed heart rate variability: A study in patients with mild-to-moderate heart failure. Chest.

[56] Chung MK, Gulik TS, Rotondo RE (1990). Mechanisms of action of cytokine inhibition of β-adrenergic agonist stimulation of cyclic AMP in rat cardiac myocytes: impairment of signal transduction. Circ Res.

[57] Mann DL, Kent RL, Parsons B (1992). Adrenergic effects on the biology of the adult mammalian cardiomyocyte. Circulation.

[58] Godin PJ, Buchman TG (1996). Uncoupling of biological oscillators: A complementary hypothesis concerning the pathogenesis of multiple organ dysfunction syndrome. Crit Care Med.

[59] Kennedy H (1998). Heart rate variability - a potential, noninvasive prognostic index in the critically ill patient. Crit Care Med.

[60] Tateishi Y, Oda S, Nakamura M (2007). Depressed heart rate variability is associated with high IL-6 blood level and decline in blood pressure in septic patients. Shock.

[61] Goldberger AL, Amaral LAN, Hausdorff JM, Ivanov PC, Peng CK, Stanley HE (2002). Fractal dynamics in physiology: alterations with disease and aging. Proc Natl Acad Sci USA.

[62] Griffin MP, Lake DE, Bissonette EA, Harrell FE, Micheal O’ Shea T, Moorman JR (2005). Heart rate characteristics: novel physiomarkers to predict neonatal infection and death. Pediatrics.

[63] Janousek O, Ronzhina M, Kolarova J, Provaznik I, Flanova K, Novakova M (2010). Heart rate variability parameters in isolated rabbit hearts; Proceedings of biosignal 2010. Analysis of biomedical signals and images.

[64] Verkerk AO, Wilders R, Coronel R, Ravesloot JH, Verheijck EE (2003). Ionic remodeling of sinoatrial node cells by heart failure. Circulation.

[65] Fairchild KD, Saucerman JJ, Raynor LL (2009). Endotoxin depresses heart rate variability in mice: cytokine and steroid effects. Am J Physiol Regul Integr Comp Physiol.

[66] Zorn-Pauly K, Pelzmann B, Lang P (2007). Endotoxin impairs the human pacemaker current I_F_. Shock.

[67] Khaykin Y, Dorian P, Tang A (1998). The effect of sinus node depression on heart rate variability in humans using zatebradine, a selective bradycardic agent. Can J Physiol Pharmacol.

[68] Joannides R, Moore N, Iacob M (2005). Comparative effects of ivabradine, a selective heart rate-lowering agent, and propranolol on systemic and cardiac haemodynamics at rest and during exercise. Br J Clin Pharmacol.

[69] Kruger C, Landerer V, Zugck C, Ehmke H, Kubler W, Haass M (2000). The bradycardic agent zate-bradine enhances baroreflex sensitivity and heart rate variability in rats after myocardial infarction. Cardiovasc Res.

[70] Noble D, Denyer JC, Brown HF, DiFransesco D (1992). Reciprocal role of the inward currents ib, Na and if in controlling and stabilizing pacemaker frequency of rabbit sino-atrial node cells. Proc R Soc Lond B Biol Sci.

[71] Pape HC (1994). Specific bradycardic agents block the hyperpolarization-activated cation current in central neurons. Neuroscience.

[72] Fox K, Ford I, Steg PG, Tendera M, Ferrari R on behalf of the
BEAUTIFUL investigators (2008). Ivabradine for patients with stable
coronary disease and left-ventricular systolic dysfunction (BEAUTIFUL):
a randomized, double-blind, placebo-controlled trial. Lancet.

[73] Fox K, Ford I, Steg PG, Tendera M, Robertson M, Ferrari R (2008). Heart rate as a prognostic risk factor in patients with coronary disease and left-ventricular systolic dysfunction (BEAUTIFUL): a subgroup analysis of a randomized controlled trial. Lancet.

[74] Bohm M, Swedberg K, Komajda M (2010). Heart rate as a risk factor in chronic heart failure (SHIFT): the association between heart rate and outcomes in a randomized placebo-controlled trial. Lancet.

[75] Zhoo ZZ, Wurster RD, Qi M, Jones SB (1991). Sympathoadrenal activation in sinoaortic denervated rats following endotoxin. Am J Physiol.

[76] Jones SB, Romano FD (1984). Plasma catecholamines in the conscious rat during endotoxicosis. Circ Shock.

[77] Palsson J, Ricksten SE, Delle M, Lundin S (1988). Changes in renal sympathetic nerve activity during experimental septic and endotoxin shock in conscious rats. Circ Shock.

[78] Jones SB, Kotsonis P, Majewski H (1994). Endotoxin enhances norepinephrine release in the rat by peripheral mechanisms. Shock.

[79] Werdan K, Schmidt H, Ebelt H, Zorn-Pauly K (2009). Impaired regulation of cardiac function in sepsis, SIRS and MODS. Can J Physiol Pharmacol.

[80] Bernardin G, Strosberg AD, Bernard A, Mattei M, Marullo S (1998). Beta-adrenergic receptor-dependent and independent stimulation of adenylate cyclase is impaired during severe sepsis in humans. Intensive Care Med.

[81] Goldstein B, Kempski MH, Stair D (1995). Autonomic modulation of heart rate variability during endotoxin shock in rabbits. Crit Care Med.

[82] Wearden PD (1999). Alterations in intrinsic heart rate in endotoxemia.
PhD dissertation.

[83] Lubbe AS, Harris PD, Garrison RN (1993). Systemic hemodynamic and microvascular responses in spontaneously hypertensive rats during *Escherichia Coli* bacteremia. Circ Shock.

[84] Dunn DL (1994). Gram-negative bacterial sepsis and sepsis syndrome. Surg Clin North Am.

[85] Harri M, Kuusela P (1982). Effects of the adrenergic nervous system on training-induced cardiac enlargement and on the intrinsic rate and phenylephrin sensitivity of isolated atria. Can J Physiol Pharmacol.

[86] Walsh RR (1969). Heart rate and its neural regulation with rising body temperature in anesthetized rats. Am J Physiol.

[87] Sage MD, West EJ, Gavin JB (1985). Cardiac performance of isolated beating heart obtained from rats anesthetized by three different agents. Lab Anim Sci.

[88] Nuding S, Werdan K, Ebelt H, Vincent JL (2011). Heart rate as prognostic marker and therapeutic target in MODS. Annual update in
intensive care and emergency medicine 2011.

[89] Muller-Werdan U, Buerke M, Ebelt H (2006). Septic cardiomyopathy-A not yet discovered cardiomyopathy?. Exp Clin Cardiol.

[90] Parker MM, Shelhamer JH, Natanson C, Alling DW, Parrillo JE (1987). Serial cardiovascular variables in survivors and nonsurvivors of human septic shock: heart rate as an early *predictor* of prognosis. Crit Care Med.

[91] Nuding S, Ebelt H, Hoke RS, Krummenerl A, Wienke A, Muller-Werdan U, Werdan K (2011). Reducing elevated heart rate in patients with multiple organ dysfunction syndrome by the If (funny channel current) inhibitor ivabradine. Modify trial. Clin Res Cardiol.

[92] Schmidt H, Saworski J, Werdan K, Muller-Werdan U (2007). Decreased beating rate variability of spontaneously contracting cardiomyocytes after co-incubation with endotoxin. J Endotoxin Res.

[93] Wondergem R, Graves BM, Ozment-Skelton TR, Li C, Williams DL (2010). Lipopolysaccharides directly decrease Ca^2+^ oscillations and the hyperpolarization-activated non-selective cation current I_f_ in immortalized HL-1 cardiomyocytes. Am J Physiol Cell Physiol.

[94] Klöckner U, Rueckschloss U, Grossmann C (2011). Differential reduction of HCN channel activity by various types of lipopolysaccharide. J Mol Cell Cardiol.

[95] Shi W, Wymore R, Yu H (1999). Distribution and prevalence of hyperpolarization-activated cation channel (HCN) mRNA expression in cardiac tissues. Circ Res.

[96] Deutschman CS, Harris AP, Fleisher LA (1994). Changes in heart rate variability under propofol anesthesia. Anesth Analg.

[97] Galletly DC, Westenberg AM, Robinson BJ (1994). Effect of halothane, isoflurane and fentanyl on spectral components of heart rate variability. Br J Anaesth.

[98] Zhang Q, Huang A, Lin YC, Yu HG (2009). Associated changes in HCN2 and HCN4 transcripts and If pacemaker current in myocytes. Biochim Biophys Acta.

[99] Rudiger A, Singer M (2007). Mechanisms of sepsis-induced cardiac dysfunction. Crit Care Med.

[100] Celes MRN, Torres-Duenas D, Alves-Filho JC (2007). Reduction of gap and adherens junction proteins and intercalated disc structural remodeling in the hearts of mice submitted to severe cecal ligation and puncture sepsis. Crit Care Med.

